# Longitudinal analysis of the association between removal of dental amalgam, urine mercury and 14 self-reported health symptoms

**DOI:** 10.1186/1476-069X-13-95

**Published:** 2014-11-18

**Authors:** Jennifer D Zwicker, Daniel J Dutton, John Charles Herbert Emery

**Affiliations:** School of Public Policy, University of Calgary, Calgary, AB T2P 1H9 Canada; Department of Economics, University of Calgary, Social Sciences Building, Room 554, 2500 University Dr. NW, Calgary, AB T2N 1 N4 Canada

## Abstract

**Background:**

Mercury vapor poses a known health risk with no clearly established safe level of exposure. Consequently there is debate over whether the level of prolonged exposure to mercury vapor from dental amalgam fillings, combining approximately 50% mercury with other metals, is sufficiently high to represent a risk to health. The objective of our study is to determine if mercury exposure from amalgam fillings is associated with risk of adverse health effects.

**Methods:**

In a large longitudinal non-blind sample of participants from a preventative health program in Calgary, Canada we compared number of amalgam fillings, urine mercury measures and changes in 14 self-reported health symptoms, proposed to be mercury dependent sub-clinical measures of mental and physical health. The likelihood of change over one year in a sample of persons who had their fillings removed was compared to a sample of persons who had not had their fillings removed. We use non-parametric statistical tests to determine if differences in urine mercury were statistically significant between sample groups. Logistic regression models were used to estimate the likelihood of observing symptom improvement or worsening in the sample groups.

**Results:**

At baseline, individuals with dental amalgam fillings have double the measured urine mercury compared to a control group of persons who have never had amalgam fillings. Removal of amalgam fillings decreases measured urine mercury to levels in persons without amalgam fillings. Although urine mercury levels in our sample are considered by Health Canada to be too low to pose health risks, removal of amalgam fillings reduced the likelihood of self-reported symptom deterioration and increased the likelihood of symptom improvement in comparison to people who retained their amalgam fillings.

**Conclusions:**

Our findings suggest that mercury exposure from amalgam fillings adversely impact health and therefore are a health risk. The use of safer alternative materials for dental fillings should be encouraged to avoid the increased risk of health deterioration associated with unnecessary exposure to mercury.

**Electronic supplementary material:**

The online version of this article (doi:10.1186/1476-069X-13-95) contains supplementary material, which is available to authorized users.

## Background

Due to the potential health risks associated with exposure to mercury, the use of dental amalgam fillings remains a source of controversy. Exposure to mercury vapor is a known health risk with no clearly established safe level of exposure [1]. Dental amalgam fillings are a major source of mercury exposure in the general population, with several studies showing a correlation between the number of amalgam surfaces and brain, blood and urine concentrations of mercury [2–9]. The continued widespread use of amalgams in dental restorations may have negative health consequences in the Canadian population. As alternative restorative materials to amalgam exist, continued use of amalgam represents an unnecessary exposure to toxicity risk from mercury. Health Canada’s 1996 Position Statement on Dental Amalgam assessed, however, that there was not sufficient evidence to support a ban on the use of amalgam fillings or the removal of sound amalgam fillings in patients who have no indication of adverse health effects attributable to mercury exposure [10].

Amalgam dental fillings have been an accepted part of restorative dentistry for over a century [11, 12], with little change to the powder comprised of approximately 43 to 50.5% mercury by weight with other metals typically including silver (40 to 70%), tin (12 to 30%), copper (12 to 24%), indium (0 to 4%), palladium (0.5%) and zinc (0 to 1%) [13]. Mercury has an important role in amalgam composition due to its unique property of being the only metal that is in the liquid phase at room temperature [14]. The widespread use of amalgam fillings can be attributed to their being easily prepared, relatively inexpensive and highly durable [15]. Mercury vapor is continuously released from amalgam restorations, the rate of which depends on the number of amalgam surfaces, location of the amalgam surfaces, age, eating/chewing habits and amalgam composition and condition [16, 17]. Mercury is a highly toxic metal, 80% of which is absorbed when inhaled and carried by the blood to cells in all organs of the body [18]. Typically mercury vapor is absorbed by the body and oxidized to ionic mercury (mercuric form Hg^++^), which can covalently bind with cell proteins [19]. This means almost any protein can be damaged if sufficient levels of mercury are present. Mercury is cytotoxic, neurotoxic, immunotoxic, and nephrotoxic, as occupational exposure to high doses of elemental mercury has been shown to impact the immune, renal and nervous system [20]. The central nervous system (CNS) is a sensitive and critical target organ due to the ability of elemental mercury to cross the blood brain barrier and access the CNS [21]. Mercury has a high affinity for selenoproteins, which are important for decreasing oxidative stress [22]. The effects of mercury on neural tissue are diverse at these levels of exposure and can include mood changes, memory and concentration problems, headache, fatigue, reduction in hand steadiness, and manual dexterity [17, 23–28].

While it is clear that mercury vapor is toxic, there is debate over whether the level of exposure resulting from dental amalgam fillings is sufficiently high to produce toxic effects in the body. The World Health Organization states “mercury may have no threshold below which some adverse effects do not occur” [1]. Other groups like the Human Biomonitoring Commission (HBM) of the German Federal Environment Agency determined that published scientific evidence and expert opinion demonstrates that there “is no risk for adverse health effects and, consequently, no need for action” for the level of mercury exposure from dental amalgam below the HBM-I level (5 μg/g-creatinine) [29]. The HBM-II level of 20 μg/g-creatinine is suggested as a level for increased risk of adverse health effects. Urine mercury levels from dental amalgam fillings are typically under 3 μg/g-creatinine, and toxicity symptoms from mercury exposure have only been consistently shown in the range of 23 to 75 μg/g-creatinine in occupational settings, with some additional evidence of toxicity symptoms in the range of 2.1 to 22 μg/g-creatinine for dental professionals working with dental amalgam materials [20, 30, 31]. These reference levels of exposure are reflective of acute, short term exposure to mercury associated most often with occupational exposure and may not be relevant for assessing the prolonged, low dose exposure from dental amalgam. Safety levels remain undefined for long-term, low-dose mercury exposure. A study by Richardson [32] translated the dose associated with amalgam exposure to compare to the Canadian chronic reference exposure level (REL) and found that the maximum number of amalgam surfaces was 4.4 and 7.3 for females and males respectively, before exceeding the Health Canada REL for Hg^0^ of 0.011 μg/kg-day. Given the estimated 17 year half-life of mercury in the brain [16, 18, 33–35], there is also concern that urine mercury levels are not reflective of the mercury burden in the CNS and some of the subsequent symptoms reported from chronic exposure.

Given the controversy over the potential adverse effects of prolonged exposure to low doses of mercury vapor from amalgam fillings, our study looks at whether the removal of amalgam fillings is associated with health changes compared to a control group of persons who did not have their fillings removed. We interpret significant associations between amalgam removal and symptom change as reflective of risks to health associated with dental amalgam. Based on definitions of reference levels for exposure like HBM, we interpret the existence of increased risk at any level of mercury as relevant to regulatory decision making in health as opposed to demonstration of sufficient damage to health. Dental amalgam fillings have unique regulatory treatment in Canada because their use predates the enactment of safety regulation for medical devices (which includes dental amalgam) [10]. Where medical devices must be shown to be safe (absent of harm or risk) before they can be approved for use, dental amalgam can remain in use until there is evidence of risk to health.

We use a longitudinal sample of participants in a preventative health program in Calgary, Alberta. Measures from the sample include the number of amalgam filling surfaces, urine mercury (μg/g-creatinine) and self-reported health symptoms proposed to be both mercury dependent and reflect sub-clinical measures of mental and physical health [36]. In addition, a number of participants chose to have their dental amalgams removed allowing for comparisons over at least 6 months’ time between a group who never had amalgam fillings (never amalgam group) with the samples of individuals who had their amalgams removed (treatment group) or those who did not have their amalgams removed (positive group).

## Methods

Our data set provides information on participants in the Pure North S’Energy Foundation program (henceforth “Pure North”), which is a philanthropic wellness and chronic-disease prevention program based in Calgary, Alberta, Canada. Participants from the general public self-selected into the Pure North program, which assesses participant health status using questionnaires, biometric measurements and laboratory tests to provide personalized preventive health care services. Healthcare workers at Pure North (including nurses, doctors, and dentists) provide participants with lifestyle counselling, dietary supplementation with vitamins, minerals and other nutrients and dental care.

There are three identifiable groups in our analysis. The first group is the “treatment group”, those individuals who had their amalgam fillings completely removed. To be included in our analysis for urine mercury changes, participants who had their amalgam fillings removed also had to have a post-removal mercury measure at least six months after the fillings were removed. The second group consists of individuals who have a positive number of amalgam surfaces throughout the study period and who have a second urine mercury measure at least six months after their first measure, referred to as “positive amalgam group”. The third group is referred to as the “never amalgam group” and is used to compare our study groups to the baseline urine mercury level of a sample of Pure North participants who have never had amalgam restorations. Our ability to compare our study groups to persons in the Pure North program who have never had amalgams is limited to baseline mercury levels. Change in mercury was not assessed in the never amalgam group because individuals with no amalgam fillings and normal mercury levels were not retested in the Pure North program, thus there were very few individuals who never had amalgams with two mercury measures in the dataset.

There was no cost to the majority of participants in our sample for the services offered, including dental care. Our sample includes individuals in the Pure North program between September 2010 and August 2013. Participants enrolled after January 2013 with an income over $25000 were required to pay a fee for the Pure North program ranging from $600- 3000 depending on the individual’s income. This involved 6 people in the urine mercury measure group and 5 people in the self-reported symptom group. All dental care was cost-shared between dental insurance and Pure North, with no cost to the individual. Program delivery was the same for all participants.

The data gathering process employed by Pure North with respect to urine mercury and amalgam surfaces has been described in detail elsewhere [37]. Briefly, participants in the Pure North program self-selected into the program and had routine biomarker measurements and provide survey reports on their wellness as part of their personalized health program. All participants in the study received lifestyle counseling and bio-detoxification supplements including: N-Acetyl-Cysteine (NAC), Alpha-Lipoic Acid (ALA) and a high potency multivitamin mineral supplement. Health care workers administered all tests. As part of the health assessment all Pure North participants had the number of dental amalgam surfaces assessed and all participants were offered the option to test their urine mercury levels. All participants with amalgam fillings were offered the opportunity to have their amalgam fillings removed by a dedicated dental team at a state-of-the art facility for the safe removal of mercury amalgam fillings and dental restoration, with extensive precaution to minimize exposure to mercury vapor and amalgam particles. Dental benefits and risks were discussed during the initial consultation. Informed consent was obtained prior to amalgam removal. Participation in the Pure North program was not contingent on amalgam removal.

There are two parts to our analysis. First, we compare urine mercury levels in the treatment and positive dental amalgam groups at baseline and after at least 6 months post amalgam removal, or after starting the Pure North program. We also compared urine mercury level of these groups to the baseline urine mercury level of a sample of participants who never had amalgam fillings. Second, we compare changes in 14 self-reported symptoms after at least one year for those in the treatment and positive amalgam groups to assess if health symptoms are more likely to improve, and not worsen, after amalgam removal. The symptoms are sub-clinical measures of mental and physical health that have been proposed as mercury dependent. We interpret the odds ratios as reflective of risks to health associated with dental amalgam. Based on definitions of reference levels for exposure like HBM, we interpret the existence of increased risk at any level of mercury as relevant to regulatory decision making in health.

### Data collection

A dentist recorded each individual’s number of amalgam surfaces after visual inspection and x-rays. Age, sex, weight, and symptom severity were gathered from routinely administered surveys and medical professional consultations. Mercury levels were measured from a sample provided by consenting participants at an appointment. The urine samples were sent by priority shipping to Doctor’s Data, Inc. (St. Charles, IL). On receipt, aliquots of each creatinine sample were made and the remaining sample was acidified to preserve mercury. Creatinine measurement was performed using a modified Jaffe method on a Beckman Coulter AU680. Samples were prepared for elemental testing based on creatinine level and measured using ICP-MS (Perkin Elmer DRCII) [38]. Urine mercury concentrations are reported as μg/g-creatinine to reduce error introduced by variation in sample volume. Urine samples were used as they are a valid measure of body burden from long term constant exposure to elemental mercury [39].

### Variables & statistical analysis

We use the number of amalgam surfaces rather than a count of amalgam fillings because there can be several amalgam surfaces per filled tooth releasing mercury vapor (typical molars have five surfaces, and on average, there are 2 amalgam surfaces per filled tooth) [32]. Some of the observations on total amalgam surfaces differed between baseline and at one year in the program. Individuals in the positive amalgams group, with differences in number of amalgams surfaces greater than 5, were excluded from the study sample. Individuals whose total surfaces did not change by more than 5 were retained as members of the positive amalgams group. It is possible that those individuals with total surface changes smaller than 5 had a constant and positive number of amalgams, but the recorded values are measured with error because of differences between those inspecting the amalgams, the way the amalgams were inspected (x-ray versus by sight), or possible transcription errors (that would be present in any administrative data set).

To be included in the analysis of a sample of self-reported symptoms that could indicate mental or physical health changes related to exposure to mercury, individuals did not need to provide a mercury measure. All variables for the symptom analysis were reported on a scale of 1 to 10, with 10 representing often experiencing the symptom and 1 representing never experiencing the symptom. Respondents are instructed that “your responses indicate how you feel today”. The 14 symptoms reported are: headaches or migraines; memory loss; depression; fatigue or sleep disturbance; anxiety; being unusually moody; confusion; stomach problems; experiencing loss of sense of smell or taste; shakiness in hands; parasthesia; unintentionally dropping things; coordination problems and muscle weakness.

For the comparison of urine mercury levels we conducted a non-parametric Kruskal-Wallis equality-of-populations rank test with Dunn’s multiple comparison post hoc test and Bonferroni correction to compare the distributions of our sample across groups. We present box plots to show the distribution of mercury in the groups. A Wilcoxon signed-rank test was used for inter-group comparison or comparison of two related measures in the treatment and positive amalgam groups. Self-reported symptoms are reported using an ordinal scale, where we consider within-individual changes in symptom score to indicate improvement (or worsening) versus a lack of improvement (or worsening) in the symptom. We define decreases in symptom scores at least one year from baseline as symptom improvements and increases in scores at least one year from baseline as symptom worsening. The positive amalgam group symptom change compared the reported symptom scores at least one year after the first symptom score report and the treatment group compares the symptom scores at entry to the Pure North program and at least one year following amalgam removal. We then estimate the odds of observing symptom improvement (or worsening) in the treatment group and the positive amalgam group using logistic regression models. Odds ratios are reported with respect to having amalgams removed, controlling for age and sex.

The variables used in this analysis were summarized with means and standard deviations (SD) for continuous variables and percentages for binary or categorical variables. Unless otherwise stated, for tests of significance, we used a significance level of p ≤ 0.05 to determine evidence of an association. All analyses were conducted in Stata 13. Ethics approval for this study was obtained from the University of Calgary (Ethics ID E-24890).

## Results

Characterization of the age, sex and urine mercury levels of the treatment, positive amalgam and never amalgam groups are shown in Table 1. Females were more represented in the positive amalgam and treatment groups with 60% and 57% females respectively. The never amalgam group was 33% female. The never amalgams group was younger (mean age 46.7 years) than the treatment group or the positive amalgam group (53.7 and 54.3 years). The positive amalgam group had a higher mean number of amalgam surfaces (23.7 surfaces) than the treatment group (18.4 surfaces) (Wilcoxon signed-rank test, p <0.05).

**Table 1:** **Summary data for study sample with urine mercury measures**

	Treatment group (N=250)	Positive Amalgam group (N=167)	Never Amalgam group (N=538)
% Female	57%	60%	33%
Age	53.7 (9.5)	54.3 (9.0)	46.7 (14.3)
Baseline Hg Overall	1.61 (1.27)	1.67 (1.64)	0.78 (1.43)
Baseline Hg Males Only	1.18 (0.97) [107]	1.42 (1.72) [67]	0.54 (0.77) [358]
Baseline Hg Females Only	1.92 (1.37) [143]	1.85 (1.56) [100]	1.24 (2.14) [180]
% above HBM-1 (proportion)	3.2% (8/250)	3.0% (5/167)	0.9% (5/538)
Number of Amalgam Surfaces at Baseline	18.4 (12.9)	23.7 (13.8)	0 N/A

Baseline values of average urine mercury measures (μg/g-creatinine) overall, for males and females across all amalgam groups. Percent above the established HBM-I risk level of 5 μg/g-creatinine overall for each group. Arithmetic mean (standard deviation) [number of observations], unless otherwise specified.

**Figure 1:**
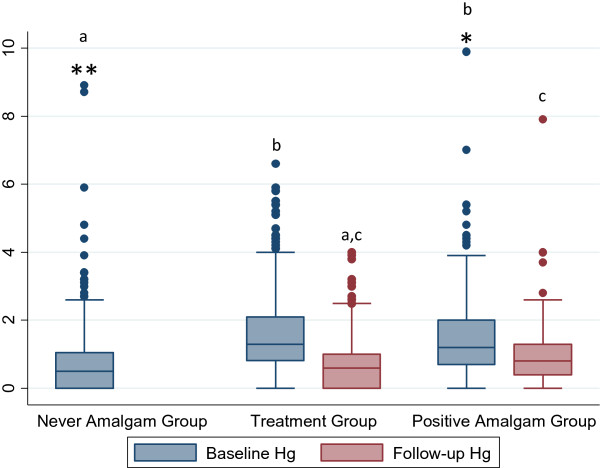
**Comparison of the baseline and follow-up urine mercury measures for treatment and positive amalgam groups.** Box and whisker plots showing distributions of urine mercury concentrations for each group (μg/g-creatinine). Corresponding numeric data are provided in Table 3. Boxes extend from the 25th to the 75th percentile, horizontal bars inside the boxes represent the median, whiskers extend to maximum and minimum observations within 1.5 times the length of the interquartile range above and below the 75th and 25th percentiles, respectively, and outliers are represented as circles. Group comparison was performed using the Kruskal-Wallis with Dunn’s multiple-comparison test p < 0.05. A Wilcoxon signed-rank test was used for inter-group comparison and all were statistically significant (p < 0.001). Lower case letters indicates distributions that are not significantly different. Values >10 μg/g-creatinine have been removed for readability. * signifies an extreme outlier: one at 12 μg/g-creatinine in the positive group and two at 11 and 23 μg/g-creatinine in the never amalgam group.

**Figure 2:**
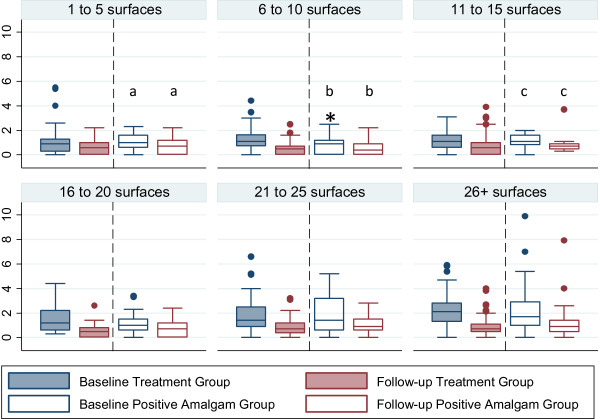
**Comparison of baseline and follow-up urine mercury concentrations grouped by number of surfaces at baseline.** Box and whisker plots showing distributions of urine mercury concentrations for the treatment group and the positive amalgam group (μg/g-creatinine). Boxes extend from the 25th to the 75th percentile, horizontal bars inside the boxes represent the median, whiskers extend to maximum and minimum observations within 1.5 times the length of the interquartile range above and below the 75th and 25th percentiles, respectively, and outliers are represented as circles. A wilcoxon signed-rank test was used for inter-group comparison and all were statistically significant except those indicated by a lower case letter (p < 0.05). Values >10 μg/g-creatinine have been removed for readability. * signifies an extreme outlier at 12 μg/g-creatinine.

Boxplots demonstrate the distribution of urine mercury for the study groups with a clear right skew (Figure 1) which has been deemed important for studying mercury exposure from dental amalgam [40]. The arithmetic mean for urine mercury concentration at baseline for the never amalgam group was 0.78 μg/g-creatinine. The mean urine mercury level of participants with one or more amalgam surfaces is about double that observed for the participants with 0 dental amalgam surfaces (1.61 μg/g-creatinine and 0.78 μg/g-creatinine respectively (Wilcoxon signed-rank test, p <0.001, Table 1 and Figure 1). Positive amalgam and treatment group baselines were not significantly different. The urine mercury level in females was consistently higher than the level in males for all groups (Kruskal-Wallis test, p <0.001, Table 2). Higher numbers of amalgam surfaces did result in higher baseline urine levels of mercury in the positive and treatment group (Figure 2). The averages in the treatment group ranged from 1.03 μg/g-creatinine for 1–5 surfaces to 2.10 μg/g creatinine for 26+ surfaces (Figure 2). A Wilcoxon signed-rank test was used for inter-group comparison between baseline and follow-up, and all were statistically significant except those indicated by a lower case letter (p < 0.05, Figure 2).

**Table 2:** **Mean urine mercury measures for treatment group and positive amalgam group**

Group		Treatment group	Positive amalgam group
		(N=250)	(N=167)
		(M: 107, F: 143)	(M: 100, F: 67)
Overall	Baseline Mercury	1.61 (1.27)	1.67 (1.64)
	Second Mercury	0.73 (0.75)	0.92 (0.90)
Males Only	Baseline Mercury	1.18 (0.97)	1.42 (1.72)
	Second Mercury	0.48 (0.48)	0.71 (0.66)
Females Only	Baseline Mercury	1.92 (1.37)	1.85 (1.56)
	Second Mercury	0.92 (0.86)	1.06 (1.01)

Mean urine mercury measures (μg/g-creatinine) for treatment group and positive amalgam group members with a second mercury measure at least six months later. Mean (standard deviation) [number of observations]. The baseline mercury measure is the first measure available for the individual (pre-removal in the treatment group). The second mercury measure is the last available mercury measure, post-removal in the treatment group.

The average urine mercury levels are low enough to be considered safe and posing no risk of adverse health effects according to the HBM values for mercury. The HBM I value (5 μg/g-creatinine) represents the concentration of a substance in human biological material below which – according to the knowledge and judgment of the commission and with regard to the substance under consideration – there is no risk for adverse health effects and, consequently, no need for action. The HBM II value (20 μg/g-creatinine) represents the concentration of a substance in human biological material above which there is an increased risk for adverse health effects and, consequently, an urgent need to reduce exposure and to provide individual biomedical care (advice) [29]. In our sample 3.2% of the treatment group, 3.0% of the positive amalgams group and 0.9% of the never amalgam group had urine mercury levels exceed the HBM-I level of safety (5 μg/g-creatinine). There was only one individual in the never amalgams group who exceeded the HBM-II level.

Removal of amalgam surfaces is associated with lower mercury levels at measurement at least six months later (Table 2). Post-removal levels of mercury in the treatment group were lower than the baseline levels overall (Wilcoxon signed-rank test, p < 0.05, Figure 1) and when broken out by number of surfaces (Wilcoxon signed-rank test, p < 0.00, Figure 2). Post-removal levels of mercury were not significantly different from the never amalgams group at baseline (Figure 1). As part of the Pure North program all of the individuals in our sample were provided with a high potency multivitamin supplement used for bio-detoxification, including NAC and ALA. Consequently, the positive amalgams group also had a reduction in urine mercury after at least six months in the program that was 85% of the reduction in the treatment group (-0.75 μg/g-creatinine compared to -0.88 μg/g-creatinine) but the difference between groups is not statistically significant (Table 2).

For each of the 14 self-reported symptoms potentially influenced by mercury exposure, we estimate logistic regression models of the odds of observing symptom improvement and symptom worsening, distinguishing between those participants who had their amalgam fillings removed and those that did not. The mean baseline self-reported symptom scores were not significantly different between the positive amalgam and treatment groups, adjusted for sex and age (Additional file 1). Odds ratios represent the increased or decreased likelihood of a symptom change occurring in the treatment group when compared to the positive amalgam group. For all 14 symptoms, the odds ratios for symptom improvement for the treatment group demonstrate greater odds of symptom improvement in comparison with the positive amalgam group (Table 3). Two of these odds ratios are significant with p ≤ 0.05 (memory loss and stomach problems) and with p ≤ 0.1 an additional 4 symptoms were significant (fatigue/sleep disturbance, confusion, loss of sense of smell or taste, shakiness in hands). Removal of fillings reduced the odds that symptoms would worsen for all 14 symptoms. Odds ratios for five symptoms (confusion, stomach problems, loss of sense of smell or taste, shakiness in hands, coordination problems) were statistically significant when p ≤ 0.05 and another two (headache, muscle weakness) when p ≤ 0.1.

**Table 3:** **One year odds of symptom improvement and worsening in the treatment group**

	Greater than one year		
Symptom	Tx Group Symptoms Improve	Tx Group Symptoms Get Worse	N
Headache	1.222 {0.342}	0.616^ {0.0516}	368
Memory loss	1.600* {0.0255}	0.721 {0.157}	374
Depession	1.206 {0.370}	0.781 {0.290}	370
Fatigue/Sleep disturbance	1.499^ {0.0598}	0.729 {0.173}	371
Anxiety	1.143 {0.522}	0.929 {0.764}	373
Unusually Moody	1.368 {0.134}	0.897 {0.649}	370
Confusion	1.462^ {0.0832}	0.613* {0.0342}	370
Stomach problems	1.571* {0.0360}	0.627* {0.0459}	369
Loss sense of smell or taste	1.588^ {0.0727}	0.376* {0.000508}	369
Shakiness in hands	1.499^ {0.0937}	0.519* {0.0102}	371
Parasthesia	1.048 {0.849}	0.840 {0.523}	345
Unintentionally dropping things	1.173 {0.481}	0.912 {0.730}	367
Coordination problems	1.122 {0.628}	0.574* {0.0329}	367
Muscle weakness	1.282 {0.246}	0.635^ {0.0676}	367

One year odds of symptom improvement and worsening in the treatment group controlling for age and sex. Odds ratio coefficients are the odds of change in treatment group relative to the positive amalgam group. {P values}; *indicates coefficient is different from 1 at size 0.05; ^represents statistical significance at size 0.10.

## Discussion

In this study individuals with dental amalgam fillings were found to have double the measured urine mercury when compared to individuals with no dental amalgam fillings. The combination of amalgam filling removal and bio-detoxification was found to reduce urine mercury levels equivalent to the never amalgam group. Our results agree with other studies that have shown that even though amalgam removal initially produces a rise in mercury levels, there is a significant reduction in urine mercury to 60-76% of the initial levels six months later [41–46].

We found similar reductions in urine mercury in the treatment and positive amalgam groups. Our results suggest that bio-detoxification reduces urine mercury levels at 85% of the reduction of combination therapy (bio-detoxification and amalgam removal). While there was a greater reduction in the treatment group versus the positive amalgam group, there was not a statistically significant difference between these groups. The near equivalence of bio-detoxification and amalgam removal for reducing urine mercury has been demonstrated in the literature [47].

It is notable that while amalgam fillings do increase exposure to mercury, our samples show levels of urine mercury considered to be safe according to current reference levels. Being below the HBM I reference level, the exposure to mercury from dental fillings would be considered to pose no risk to health and warrant no action to address. As filling removal reduces mercury levels which are already considered safe, there is also the implication that removal of fillings would result in no health benefit and if anything expose individuals to the risk of bolus exposure to mercury at the time of removal.

Our analysis of symptom change following amalgam removal challenges the preceding assessments of the safety of dental amalgam. In samples of persons with mercury exposure below the HBM I threshold, we found that the removal of amalgam fillings increased likelihood of reporting symptom improvement and decreased likelihood of self-reported symptoms worsening in comparison with those who retained their fillings. Significant improvement attributable to the amalgam removal treatment was seen for memory loss and stomach problems. Significantly less deterioration was seen for confusion, stomach problems, loss of sense of smell or taste, shakiness in hands, and coordination problems.

Our results add to the body of evidence that there is reduced likelihood of health complaints after removal of the amalgam fillings, consisting of either a decrease of subjective symptom burden overall or subtle effects on symptoms, mood, motor function (hand steadiness) and cognition [42, 45–50]. Our findings suggest that there remains increased risk of health deterioration in study participants even with mercury levels below the established HBM-I no risk urine mercury value. Subclinical adverse neurobehavioral and psychological effects have been associated with a dose–response relationship with urine mercury levels that can occur with amalgam fillings (less than 4 μg/g-creatinine) [17, 26, 51–55]. The possible dose–response relationship between low-level mercury vapor exposure and subclinical neurological and psychological effects has been characterized previously as preclinical symptoms (predominantly in dentists and dental assistants) [17, 51, 56]. Symptom changes associated with amalgam removal have been seen in other studies and include a diverse range of adverse effects including decreased motor functioning (e.g., losses in hand steadiness and manual dexterity, progressing to incoordination, imbalance and tremor in muscles that perform fine motor control) and loss of mental capacity (e.g., memory, confusion, diminished logical reasoning and concentration) [42, 45, 47, 50]. Most of these studies are limited by their small sample size (amalgam removal group sample sizes of 20, 20, 60 and 796 respectively with the latter being a retrospective study). There have been reports of an absence of symptom improvement with amalgam removal [46, 48] however, the findings from these studies are limited by treatment groups of 55 and 78 participants respectively, while our treatment group has 250 participants. It should be noted that the lack of significant neurobehavioral or motor deficits associated with low level mercury exposure seen in two randomized control trials in children [57, 58], do not address the effects that early toxic exposure to mercury may have on health later in life [59].

The positive influence of amalgam removal on self-reported symptom changes, suggests that bio-detoxification is not equivalent to filling removal for health outcomes despite reducing urine mercury levels (Figure 1). Bio-detoxification and chelation therapies have been proposed as potential treatments for health conditions associated with dental amalgams [47, 50]. Bio-detoxification may have affected mercury excretion so that urine mercury is not an accurate measure of the mercury present and accumulated in the body. Typically urine is a good measure of mercury from amalgams, as urine is not a primary excretion route for methyl mercury and urine measures reflect cumulative exposure over 2 to 4 months [60]. However, the percentage of total daily mercury excretion by the urine route can range from 10% to 40%, depending on the amalgam filling exposure, and it is unclear how bio-detoxification affects this [61].

It is important to note that we are observing health impacts of amalgam fillings in persons who would not be expected to be at risk of health problems from amalgam fillings. High levels of mercury in the diet, an allergy to mercury, impaired kidney function and environmental or workplace exposure to mercury are all contraindications for dental amalgams according to Health Canada [62, 63]. We have observed symptom changes in persons without urine mercury levels associated with dietary, environmental or occupational exposures considered to be harmful. The improvements we see are likely unrelated to allergy/hypersensitivity where symptoms are clear and as a consequence those members of the population would only be included in our study in the never amalgam group.

Ultimately, there is concern that long-term exposure to low levels of mercury can contribute to neurodegenerative disorders such as Alzheimer’s disease [64]. The CNS is a sensitive target organ for mercury vapor exposure, with the most consistent and pronounced effects [21, 65], although little is known about the distribution pattern in the human brain. It is estimated that adults are typically exposed to mercury from amalgam fillings at approximately 2 to 5 μg/ day and mercury has a significantly longer half-life (over 17 years) in the brain [16, 18, 33–35]. Autopsy studies have shown that elevated brain mercury levels are correlated with number of amalgam fillings and individuals with more than 12 amalgam fillings have more than 10-times higher mercury levels in several tissues including the brain, compared to individuals with only 0–3 amalgam fillings [66, 67]. However there have been no clear clinically defined deficiencies with these levels of mercury exposure. Given this information, the subclinical symptoms experienced may be a reflection of the low level continual dosing occurring from amalgam fillings. Our results suggest that removal of the fillings may stop this dosing from occurring, but ultimately long term follow-up studies are needed to investigate this. In the meantime, given that mercury is highly neurotoxic and there is no established benefit at any level of mercury, removing amalgam fillings to prevent future buildup of mercury in the brain may be important to reduce the risk of health deterioration.

An alternative interpretation of our findings is that the removal of dental fillings was based on the initiative of the patients resulting in a non-random selection of participants in the treatment group. Individuals in the treatment group may report improvement based on more optimistic expectations or gratitude in relation to having received the intervention. Blinding was not possible in this study, as replacement of amalgam fillings was not masked. There is evidence that the placebo effect from sham surgery can last for over 12 months post-surgery [68, 69]. Participants in the treatment group may have had their concern alleviated regarding possible adverse symptoms associated with amalgam fillings (nocebo effect) [70]. We suggest that this bias is unlikely to impact the urine mercury measures and is isolated to the self-reported symptoms reporting. While we cannot rule out this bias, we suggest that it is unlikely as this bias would require a significant proportion of the treatment and positive amalgam group to improve or not improve respectively their answers to the questions a year later. The self-reported symptom questions were embedded as a part of an extensive health and wellness questionnaire and were not asked in the context of amalgam removal. If this bias is present, the positive amalgam group would also have increased odds of symptom improvement and decreased odds of symptom worsening, based on their belief that the Pure North diet, lifestyle and bio-detoxification counselling helped them.

Our findings are limited by what some will consider a short time of observation, reliance on subjective, self-reported health symptoms and lack of a control population for comparison of changes over time. It is possible that a longer follow-up period would provide different symptom effects. A recent study characterized a reduction of health complaints in persons 3 years after replacement of dental amalgams in comparison to complaints from the general population [45]. This emphasizes the magnitude of health complaints attributed to dental amalgams that can be reduced simply by removal of amalgam fillings. However, the lack of direct correlation of symptom improvement with mercury measures is a limitation of this and other studies [42]. Further, controversy exists with other studies that have failed to find a positive correlation between amalgam fillings and adverse self-reported symptoms [71–73]. This can be due at least in part, to the heterogeneity of patients with health complaints attributed to amalgam fillings, with complaints ranging from local intra-oral complaints to multiple general systemic complaints. There is a spectrum of sensitivity to mercury such that those most sensitive to mercury exposure receive greater benefit from amalgam filling removal [74].

## Conclusions

Mercury vapor poses a known health risk with no clearly established safe level of exposure [1]. Amalgam dental fillings are one of the largest sources of exposure to mercury in the general population. Our study shows individuals with dental amalgam fillings have double the measured urine mercury compared to a control group of persons who have never had amalgam fillings. Removal of amalgam fillings in persons with urine mercury levels, considered by Health Canada to be too low for adverse health effects, decreases measured urine mercury to levels in persons without amalgam fillings and reduces the odds of deterioration in self-reported health symptoms compared to a sample of persons who did not have their fillings removed within the one year timeframe. The likelihood of symptom improvement in comparison to people who retained their amalgam fillings was also increased. Health Canada’s position statement on amalgam removal indicated that there was not sufficient evidence of adverse health effects due to mercury exposure to support a total ban of amalgam or removal of amalgams from patients. Ultimately our findings suggest that mercury could have toxic effects at low levels of exposure. The use of safer alternative materials for dental fillings should be encouraged to prevent an unnecessary risk of health deterioration associated with mercury exposure from dental fillings.

## Electronic supplementary material

Additional file 1: **Summary data and baseline values for sample population with self-reported symptoms.** (PDF 106 KB)

## Abbreviations

ALA: Alpha-lipoic acid
CNS: Central nervous system
HBM: Human biomonitoring commission
NAC: N-acetyl-cysteine
REL: Reference exposure level
SD: Standard deviation.
