# Supplementary material for: Longitudinal analysis of the association between removal of dental amalgam, urine mercury and 14 self-reported health symptoms
Source: Environ Health. 2014 Nov 18;13:95. doi: 10.1186/1476-069X-13-95 (PMC4273453; doi:10.1186/1476-069X-13-95)
Supplement: Supplementary file 1 — Additional file 1: Summary data and baseline values for sample population with self-reported symptoms. (PDF 106 KB) [file 12940_2014_811_MOESM1_ESM.pdf]

Appendix 1.Summary data and baseline values for sample population with self-reported symptoms

	Overall	Treatment Group	Positive Amalgam Group
**Summary Data**			
% Female	52%	54%	49%
Age	48.6 (10.5)	48.5 (10.0)	48.7 (11.0)
Number of Amalgam Surfaces at Baseline	22.21 (15.58)	21.19 (16.43)	23.34 (14.53)
**Baseline Self-Reported Symptom Scores**			
Headache	3.85 (2.39)	3.86 (2.40)	3.83 (2.38)
Memory Loss	4.62 (2.56)	4.56 (2.41)	4.69 (2.73)
Depression	3.90 (2.44)	3.82 (2.50)	3.99 (2.37)
Fatigue	5.84 (2.76)	5.91 (2.78)	5.76 (2.72)
Anxiety	4.36 (2.38)	4.36 (2.44)	4.35 (2.32)
Moody	3.72 (2.28)	3.78 (2.35)	3.66 (2.20)
Confusion	2.84 (2.25)	2.88 (2.28)	2.79 (2.22)
Stomach problems	3.98 (3.02)	4.23 (3.10)	3.71 (2.91)
Loss of sense of smell and taste	2.06 (2.05)	2.08 (1.95)	2.03 (2.16)
Hand Shakiness	2.42 (2.26)	2.41 (2.20)	2.44 (2.34)
Parasthesia	2.43 (2.39)	2.36 (2.35)	2.51 (2.43)
Dropping things	2.39 (2.21)	2.45 (2.23)	2.33 (2.18)
Coordination problems	2.25 (1.94)	2.27 (1.88)	2.22 (2.01)
Muscle weakness	3.19 (2.48)	3.23 (2.51)	3.16 (2.45)

Summary data presented for memory loss is representative for all other symptoms. Arithmetic mean and standard deviations are in parentheses unless otherwise specified, percentages where indicated.
